# Investigation of a New Stacking Pattern of Laminates with Approximately Constant Bending Stiffness

**DOI:** 10.3390/polym17081098

**Published:** 2025-04-18

**Authors:** Qingnian Liu, Yingfeng Shao, Yong Cai, Long Li, Fan Song

**Affiliations:** 1 State Key Laboratory of Nonlinear Mechanics (LNM), Institute of Mechanics, Chinese Academy of Sciences, Beijing 100190, China; liuqingnian@imech.ac.cn (Q.L.); lilong@lnm.imech.ac.cn (L.L.); 2 The System Design Institute of Mechanical—Electrical Engineering, Beijing 100854, China; 3 School of Engineering Science, University of Chinese Academy of Sciences, Beijing 100049, China; 4 Aerospace Research Institute of Materials & Processing Technology, Beijing 100076, China

**Keywords:** stacking pattern, CFRP, normalized direction factor of bending stiffness (NDFBS), constant bending stiffness, space mirrors

## Abstract

To achieve laminates with constant bending stiffness to match the high precision requirement of optical systems made of carbon fiber reinforced plastic (CFRP), a new method, the normalized direction factor of bending stiffness (NDFBS), is proposed based on the normalized geometric factor of bending stiffness. Using NDFBS and its variance (VNDFBS), we investigate two common stacking patterns, I and II ([(*θ*_1_)*_m_*/(*θ*_2_)*_m_*/…/(*θ_p_*)*_m_*]_S_ and [(*θ*_1_/*θ*_2_/…/*θ_p_*)*_m_*]_S_) and our proposed new stacking pattern, Pattern III ([(*θ*_1_/*θ*_2_/…/*θ_p_*)_S_]*_m_*) based on the initial quasi-isotropic laminates, [*θ*_1_/*θ*_2_/…/*θ_p_*]. The bending stiffness of the stacking sequence [(45/−45/0/90)_S_]_2_ tends to be more uniform than that of [45/−45/0/90]_2S_, and the order of uniformity in bending stiffness of other stacking sequences is [(60/0/−60)_S_]_4_ > [60/0/−60]_4S_ > [(60/0/−60)_S_]_2_ > [60/0/−60]_2S_. Both theoretical deviations and experimental observations confirm that as the cycle number *m* increased, the uniformity in bending stiffness is improved gradually, except for that of Pattern I. As the cycle number increased, the speed of Pattern III approaching the constant bending stiffness was faster than that of Patterns I and II_._ Notably, to achieve a nearly identical uniformity in bending stiffness, only the square root of the cycle number of Pattern II was enough for Pattern III. Based on the same initial laminate and cycle number, Pattern III exhibited more uniform bending stiffness and strength, which are appropriate for precision optical components that require dimensional stability, such as space mirrors.

## 1. Introduction

With the progress of space technology, the requirements for the accuracy of earth observation and communication are higher and higher. The space camera and telescope need a bigger diameter and higher resolution of the optical system, as well as a variety of reflectors mounted on a satellite or aircraft [1,2,3,4]. Due to the space environment’s variability and transport costs, carbon fiber reinforced plastic (CFRP) with good thermal stability, high specific modulus, and low density becomes one of the best material selections for mirrors with a larger diameter and higher precision [5,6,7,8,9].

CFRP is an orthotropic material, and the mirrors are mainly affected by an inertial force or thermal stress caused by uniform temperature change [1,2]. To effectively improve dimensional stability and the uniformity of deformation, CFRP mirrors or reflective surfaces are usually designed as a fully isotropic laminate [10,11,12,13,14]. The in-plane and out-of-plane stiffnesses of fully isotropic laminates are referred to as isotropic; that is, on a macroscopic scale, their performance is the same as that of isotropic material [15,16,17,18]. However, due to the anisotropic property of the composite material, the test and simulation results showed that the inevitable ply angle deviations in the process of cutting and stacking the prepreg would cause out-of-plane deformations of the reflection mirror during its forming and employment. Deformations generally appear as a saddle shape, which decreases the precision of mirrors [3,19,20,21]. Fortunately, mirrors made of fully isotropic laminates would significantly reduce the influence of the ply angle deviations on the profile accuracy [8,22,23].

Therefore, it is of great significance to study fully isotropic laminates, which have attracted many researchers’ attention since the 1970s [24,25]. Based on the kind of laminate, fully isotropic laminates could be classified into three categories: symmetric laminates, antisymmetric laminates, and general laminates.

The laminates above are symmetric in geometry and material properties at about the mid-plane. Then, there is no tensile and bending coupling stiffness matrix [25,26] and no thermal moment caused by uniform temperature or humidity change [14]. In general, symmetric laminates are used in conjunction with quasi-isotropic laminates. The angles between the adjacent plies are fixed, and each direction has the same number of plies [18], which have equal extensional stiffness in all directions. Regarding the symmetric and quasi-isotropic laminates, only the uniformity of bending stiffness needs to be considered. Kuang M. Wu and Bian L. Avery [17] proposed the concept of weight factor and obtained a set of quasi-isotropic laminates in bending stiffness. However, at least 36 plies were required. To decrease the number of plies, investigating the approximately constant bending stiffness of laminates has become the main research field. Pryor [10,11] and Zeng [13] suggested optimizing stacking sequences by coefficients of bending/twisting and the regularization stiffness coefficient method, respectively. Due to limiting the stacking sequences as [(*θ*_1_/*θ*_2_/…/*θ_p_*)*_m_*]_S_, their optimization results were that the more the ply angles, the better the laminates. Liu [16,27] concluded that the average of the surface peak-to-valley (PV) could be regarded as an index to estimate the consistency of bending stiffness and a rare stacking sequence, [(*θ*_1_/*θ*_2_/…/*θ_p_*)_S_]*_m_*, with *m* times cycled after once symmetry proposed to have more uniformity in bending stiffness than that of the previous stacking pattern, [(*θ*_1_/*θ*_2_/…/*θ_p_*)*_m_*]_S_. However, its theoretical proof is insufficient and lacks experimental verification, making it inconvenient.

Compared to symmetric laminates, antisymmetric laminates are usually used with symmetric and angle-ply laminates, such as [*E*/−*E*], where *E* = [(±65)/(±20)2/(±85)/(±45)]_S_ [28,29,30]. However, this kind of laminate required at least 40 plies, and their ply angles of the exact solutions were not always the integers, and the previous example was just a result after rounding. This method was always extended to seek the laminate with the special stiffness, not the constant stiffness [28,29,30,31,32,33], which limited the wide application of this method.

Concerning the general laminates, there are two primary ways. M. Grediac [34] constructed an objective function with 12 regularized stiffness factors, not 4 regularized bending stiffness factors only, for the symmetric and quasi-isotropic laminates. P. Vannucci and G. Verchery [35,36,37,38] rewrote the stiffness matrices in polar form with the transformed matrix and considered the constant stiffness in-plane and out-of-plane. There was a huge amount of computing during the optimization process of this kind of laminate, and usually, the extra restrictions were added or relaxed.

Fully isotropic laminates must be convenient to implement and decrease uncontrollable factors, which may affect the structural size and the forming accuracy. Therefore, this paper proposes the normalized direction factor of bending stiffness based on symmetric and quasi-isotropic laminates. The theoretical analysis using that method and the experiment on the effective bending stiffness proved that the stacking pattern, like [(*θ*_1_/*θ*_2_/…/*θ_p_*)_S_]*_m_*, had more uniform bending stiffness than the traditional stacking patterns. Furthermore, the same rule applies to bending strength.

## 2. Normalized Direction Factor of Bending Stiffness (NDFBS)

### 2.1. Normalized Stiffness Matrix

To efficiently compare the stiffness matrices, the extensional stiffness, *A_ij_*, and the bending stiffness, *D_ij_*, could be respectively normalized to $A_{ij}^{*}$ and $D_{ij}^{*}$ in an invariant form as [25]:

(1) $$\left\{\begin{array}{l} A_{11}^{*}\\ A_{22}^{*}\\ A_{12}^{*}\\ A_{66}^{*}\\ A_{16}^{*}\\ A_{26}^{*} \end{array}\right\}=\left[\begin{array}{ccc} U_{1} & V_{1A}^{*} & V_{2A}^{*}\\ U_{1} & -V_{1A}^{*} & V_{2A}^{*}\\ U_{4} & 0 & -V_{2A}^{*}\\ U_{5} & 0 & -V_{2A}^{*}\\ 0 & V_{3A}^{*}/2 & V_{4A}^{*}\\ 0 & V_{3A}^{*}/2 & -V_{4A}^{*} \end{array}\right]\left\{\begin{array}{l} 1\\ U_{2}\\ U_{3} \end{array}\right\}$$

(2) $$\left\{\begin{array}{l} D_{11}^{*}\\ D_{22}^{*}\\ D_{12}^{*}\\ D_{66}^{*}\\ D_{16}^{*}\\ D_{26}^{*} \end{array}\right\}=\left[\begin{array}{ccc} U_{1} & V_{1D}^{*} & V_{2D}^{*}\\ U_{1} & -V_{1D}^{*} & V_{2D}^{*}\\ U_{4} & 0 & -V_{2D}^{*}\\ U_{5} & 0 & -V_{2D}^{*}\\ 0 & V_{3D}^{*}/2 & V_{4D}^{*}\\ 0 & V_{3D}^{*}/2 & -V_{4D}^{*} \end{array}\right]\left\{\begin{array}{l} 1\\ U_{2}\\ U_{3} \end{array}\right\}$$

where $V_{iA}^{*}$ and $V_{iD}^{*}$ indicate, respectively, the normalized geometric factors of extensional and bending stiffnesses and are given by the following:

(3) $$V_{\left[1A,2A,3A,4A\right]}^{*}=\frac{1}{n}\sum_{k=1}^{n}\left[\cos2\theta_{k},\cos4\theta_{k},\sin2\theta_{k},\sin4\theta_{k}\right]\;$$

(4) $$V_{\left[1D,2D,3D,4D\right]}^{*}\;=\frac{4}{n^{3}}\sum_{k=1}^{n}[\cos2\theta_{k},\cos4\theta_{k},\sin2\theta_{k},\sin4\theta_{k}]\left[{\left(h_{k}^{*}\right)}^{3}-{\left(h_{k-1}^{*}\right)}^{3}\right]$$

(5) $$h_{k}^{*}=\frac{h_{k}}{\delta }=-\frac{n}{2}+k$$

**Figure 1:**
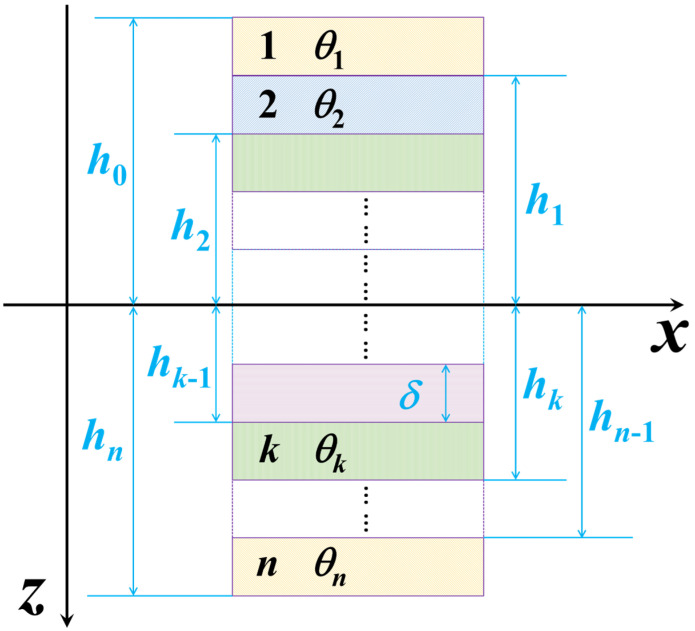
Coordinate locations of plies in a symmetric laminate. *h_k_*_−1_ and *h_k_* are the *z*-coordinates of the top and bottom surfaces with regard to ply *k*, respectively, whose orientation is denoted as *θ_k_*, and *δ* is the ply thickness.

The terms *U_i_* (*i* = 1, 2, …, 5) are invariants of the stiffness coefficients, *h_k_* is the height coordinate of the top surface of the *k*th ply, *δ* is the thickness of the ply, and *n* is the number of plies as depicted in Figure 1.

For the quasi-isotropic laminate, there are the following:

(6) $$\sum_{k=1}^{n}\cos2\theta_{k}=\sum_{k=1}^{n}\sin2\theta_{k}=\sum_{k=1}^{n}\cos4\theta_{k}=\sum_{k=1}^{n}\sin4\theta_{k}=0$$

Substitute into Equation (3) and achieve [25]:

(7) $$V_{1A}^{*}=V_{2A}^{*}=V_{3A}^{*}=V_{4A}^{*}=0$$

Thus, the quasi-isotropic laminate has constant extensional stiffness. And the necessary and sufficient conditions for the constant bending stiffness or the quasi-isotropic bending stiffness are as follows:

(8) $$V_{1D}^{*}=V_{2D}^{*}=V_{3D}^{*}=V_{4D}^{*}=0$$

### 2.2. Definition of NDFBS

Through comparing Equation (3) with Equation (4), there are two methods to satisfy Equation (8) based on the quasi-isotropic and symmetric laminates:

1. ($h_{k}^{*}$)^3^ − ($h_{k-1}^{*}$)^3^ ≡ Const. This condition is too hard to follow since the ply thickness within a laminate is generally constant or varies only slightly [39].
2. The sum of the cubic difference with the same ply orientation is constant, named the normalized direction factors of bending stiffness (NDFBS), η^*^(*j*), that is,

(9) $$\eta^{*}(j)=\frac{4}{n^{3}}\sum_{i=1}^{2m}\left[{\left(h_{a_{ij}}^{*}\right)}^{3}-{\left(h_{a_{ij}-1}^{*}\right)}^{3}\right]$$

where *a_ij_* indicates the ply number of the *i*th ply in the *j*th orientation, and there are 2*m* plies in each orientation. The NDFBS is just the normalized form of the initial direction factors [16] and is more convenient to compare and analyze. Assuming that there are *p* ply orientations, that is, the sequence number of ply orientation, *j*, ranges from 1 to *p*, then the sum of all NDFBSs follows as 1.

(10) $$\sum_{j=1}^{p}\eta^{*}(j)=\frac{4}{n^{3}}\sum_{k=1}^{n}\left[{\left(h_{k}^{*}\right)}^{3}-{\left(h_{k-1}^{*}\right)}^{3}\right]=1$$

When η^*^(*j*) ≡ 1/*p*, all NDFBSs are the same, and the laminate is the constant bending stiffness.

### 2.3. NDFBSs for Three Stacking Patterns

**Figure 2:**
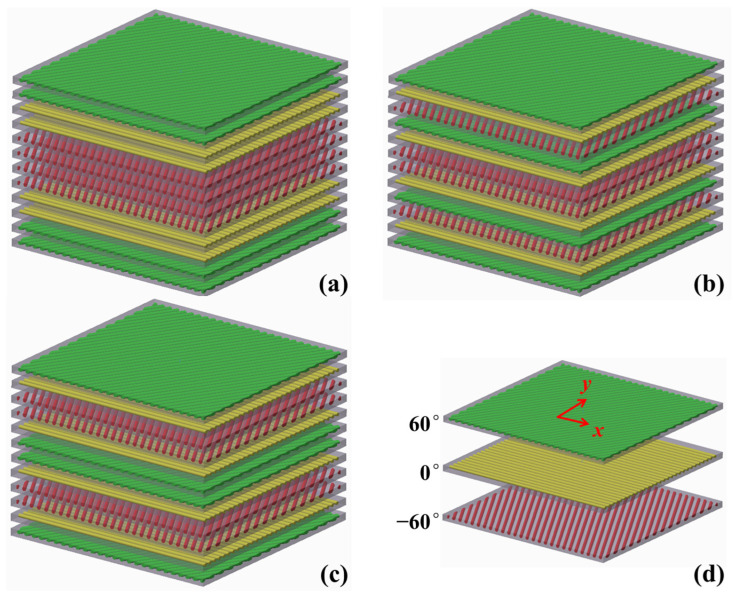
Schematic diagram of stacking patterns corresponding to three stacking patterns. (**a**) I: [(60)_2_/(0)_2_/(−60)_2_]_S_; (**b**) II: [60/0/−60]_2S_; (**c**) III: [(60/0/−60)_S_]_2_; (**d**) Legend. Green represents the 60° ply, yellow represents the 0° ply, and red represents the −60° ply.

A quasi-isotropic stacking sequence, [*θ*_1_/*θ*_2_/…/*θ_p_*], with the equal adjacent angles *π*/*p*, would be served as the basic laminate for the three stacking patterns, such as the schematic diagram in Figure 2 with *p* = 3.

#### 2.3.1. Laminates with Symmetry After Each Ply Cycle

On the basic laminate, each ply cycled *m* times before symmetry, [(*θ*_1_)*_m_*/(*θ*_2_)*_m_*/…/(*θ_p_*)*_m_*]_S_, was classified as Pattern I, as shown in Figure 2a. The ply number is as follows:

(11) $$a_{ij}=\left\{\begin{array}{ll} m(j-1)+i, & \;i=1,2,\ldots ,m;j=1,2,\ldots ,p\\ n-m(j+1)+i, & \;i=m+1,m+2,\ldots ,2m;j=1,2,\ldots ,p \end{array}\right.$$

and

(12) n=2mp

Combined with Equations (11) and (12), the NDFBS for Pattern I is simplified to the following:

(13) $$\eta_{I}^{*}(j)=\frac{1}{p}+\frac{2(p+1)(2p+1)-6j\left(2p+1-j\right)}{2p^{3}}$$

where $\eta_{I}^{*}(j)$ is independent of the cycle number, so it is easy to understand that *m* adjacent plies with the same ply orientations could be regarded as one ply.

#### 2.3.2. Laminates with Symmetry After Cycle

Similarly, the basic laminate cycled *m* times before the symmetry, [(*θ*_1_/*θ*_2_/…/*θ_p_*)*_m_*]_S_, was classified as Pattern II, as shown in Figure 2b. The ply number obeys the following:

(14) $$a_{ij}=\left\{\begin{array}{ll} (i-1)p+j, & \;i=1,2,\ldots ,m;j=1,2,\ldots ,p\\ ip+1-j, & \;i=m+1,m+2,\ldots ,2m;j=1,2,\ldots ,p \end{array}\right.$$

Combined with Equations (12) and (14), then the NDFBS for Pattern II could be obtained:

(15) $$\eta_{\mathrm{II}}^{*}(j)=\frac{1}{p}+\frac{3p\left(p+1-2j\right)}{2mp^{3}}+\frac{(p+1)(p+2)-6j\left(p+1-j\right)}{2m^{2}p^{3}}$$

#### 2.3.3. Laminates with Symmetry Before Cycle

Likewise, the basic laminate cycled *m* times after the symmetry, [(*θ*_1_/*θ*_2_/…/*θ_p_*)_S_]*_m_*, was classified as Pattern III, as shown in Figure 2c. The ply number obeys the following:

(16) $$\;a_{ij}=\left\{\begin{array}{ll} (i-1)p+j, & \;i=1,3,5,\ldots ,2m-1;j=1,2,\ldots ,p\\ ip+1-j, & \;i=2,4,6,\ldots ,2m;j=1,2,\ldots ,p \end{array}\right.$$

Combined with Equations (12) and (16), then the NDFBS for Pattern II is as follows:

(17) $$\eta_{\mathrm{III}}^{*}(j)=\frac{1}{p}+\frac{2(p+1)(2p+1)-6j\left(2p+1-j\right)}{2m^{2}p^{3}}$$

### 2.4. Comparing Three NDFBSs

**Figure 3:**
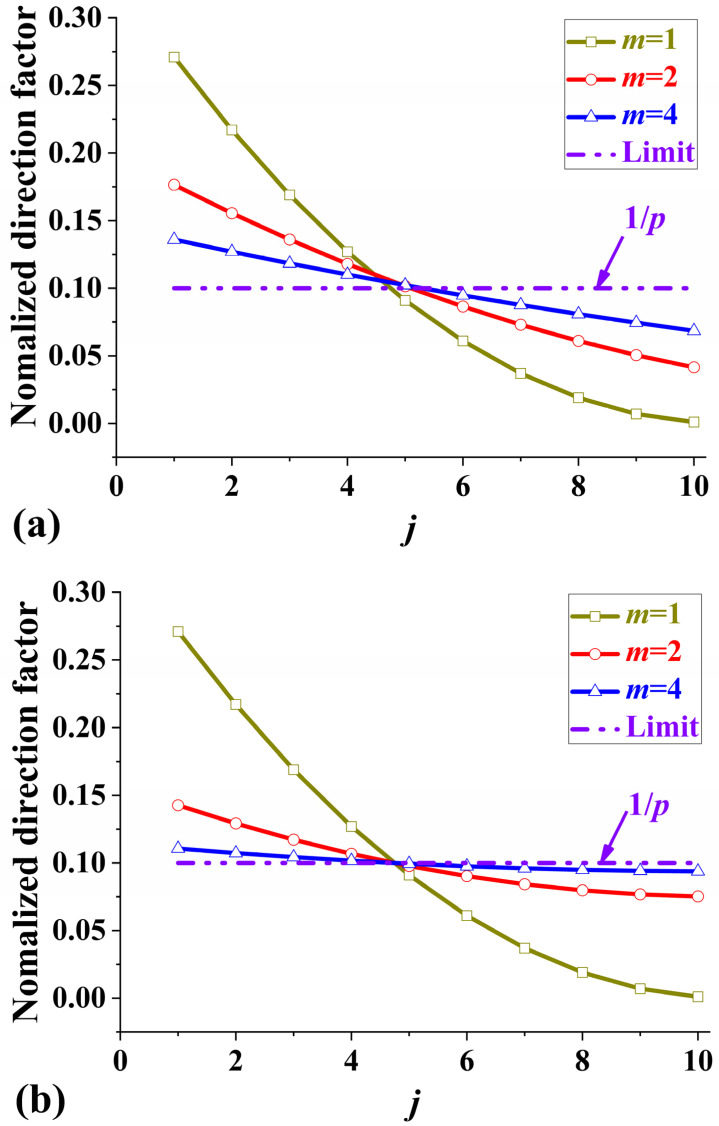
Curves of NDFBSs for different stacking patterns. (**a**) Pattern II; (**b**) Pattern III with a ply orientation of *p* = 10 and different cycle number *m*. The horizontal axis, *j*, represents the *j*th orientation.

As the cycle number, *m*, gradually grows, the NDFBSs of the Pattern II and III laminates approach the limit, 1/*p*, as shown in Figure 3. But the NDFBS of Pattern I is independent of the cycle number *m*. When *m* is equal to 1, the NDFBSs of Patterns II and III reduce to that of Pattern I,

(18) $$\eta_{I}^{*}(j){|}_{m=1}=\eta_{\mathrm{II}}^{*}(j){|}_{m=1}=\eta_{\mathrm{III}}^{*}(j){|}_{m=1}\equiv \eta_{I}^{*}(j){|}_{m}$$

Compared with the distance to the limit in Figure 3, each direction factor of Pattern III almost has a faster tendency to the limit than that of Pattern II.

### 2.5. Variance of the Normalized Direction Factors of Bending Stiffness (VNDFBS)

Considering the existence of a limit, differences between NDFBSs and the limit are flags that indicate how far away the laminate is from the constant bending stiffness or modulus. The limit of the NDFBS is equivalent to the average value of that. Therefore, the variance of the normalized direction factors of bending stiffness (VNDFBS) is as follows:

(19) $$\mu^{*}(m)=\frac{1}{p}\sum_{j=1}^{p}{\left(\eta^{*}(j)-1/p\right)}^{2}$$

μ^*^(*m*) is a function of *m*, and *p* is a parameter. To approach the laminate with constant bending stiffness, μ^*^(*m*) is required to minimize. According to the above formulas and Equations (13), (15), and (17), the VNDFBSs for the three stacking patterns could be achieved as follows:

(20) $$\mu_{I}^{*}(m)=\frac{4p^{4}-5p^{2}+1}{5p^{6}}$$

(21) $$\mu_{\mathrm{II}}^{*}(m)=\frac{15p^{4}-15p^{2}}{20m^{2}p^{6}}+\frac{p^{4}-5p^{2}+4}{20m^{4}p^{6}}$$

(22) $$\mu_{\mathrm{III}}^{*}(m)=\frac{4p^{4}-5p^{2}+1}{5m^{4}p^{6}}$$

From the above three equations, three VNDFBSs are just equivalent when *m* = 1, and that value is denoted as *f*(*p*), namely,

(23) $$f(p)=\mu_{I}^{*}(1)=\mu_{\mathrm{II}}^{*}(1)=\mu_{\mathrm{III}}^{*}(1)=(4p^{4}-5p^{2}+1)/5p^{6}$$

**Figure 4:**
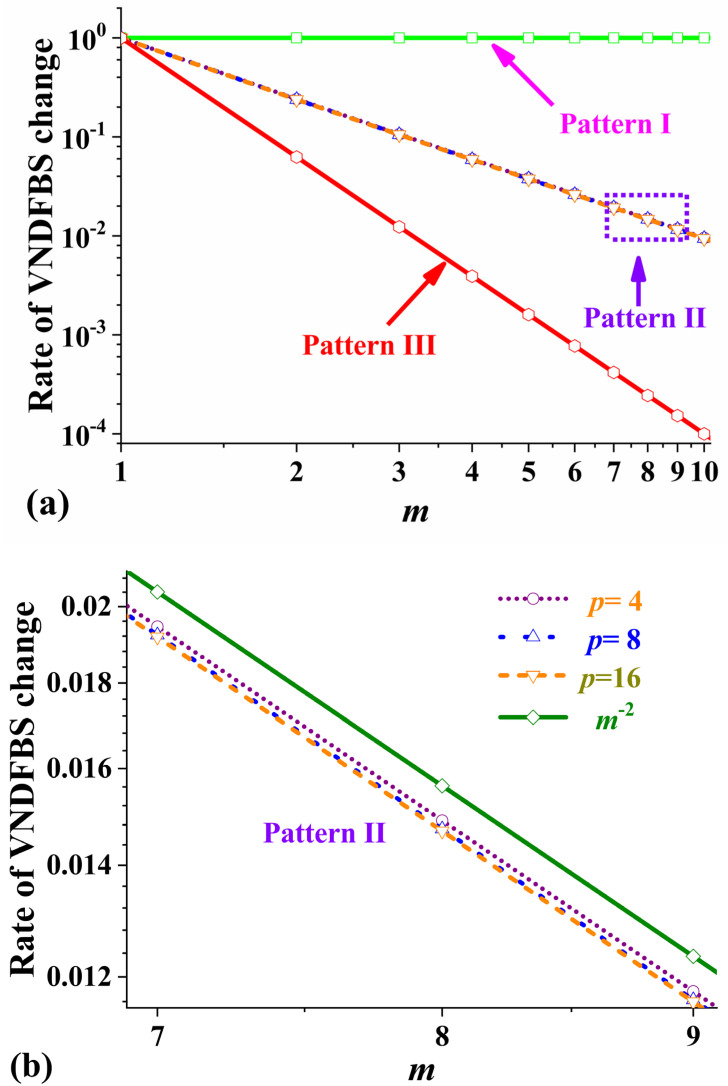
(**a**) Rate of VNDFBS change with *m* for three patterns in log–log coordinates. (**b**) Rate of VNDFBS change of Pattern II laminates with *p* = 4, 8, and 16 and the power function, *m*^−2^.

Then, Equations (20)–(22) could be rewritten as follows:

(24) $$\pi_{I}^{*}(m)=\mu_{I}^{*}(m)/f(p)=m^{0}$$

(25) $$\pi_{\mathrm{II}}^{*}(m)=\mu_{\mathrm{II}}^{*}(m)/f(p)=\frac{1}{m^{2}}\frac{15p^{2}+(p^{2}-4)/m^{2}}{15p^{2}+(p^{2}-4)}=\alpha \cdot m^{-2}$$

(26) $$\pi_{\mathrm{III}}^{*}(m)=\mu_{\mathrm{III}}^{*}(m)/f(p)=m^{-4}$$

where π^*^(*m*) is denoted as the rate of VNDFBS change, as shown in Figure 4, and is only independent of the number of ply orientations, *p*, except $\pi_{\mathrm{II}}^{*}$(*m*). Fortunately, *α*, the function of *p* and *m*, generally ranges from 0.93 to 0.98 when *p* ≥ 3 and *m* ≥ 2. $\pi_{\mathrm{II}}^{*}$(*m*) is only slightly smaller than the power function, *m*^−2^, at 8% or less, as shown in Figure 4b, and that difference is almost negligible. Then, $\pi_{I}^{*}$(*m*), $\pi_{\mathrm{II}}^{*}$(*m*), and $\pi_{\mathrm{III}}^{*}$(*m*) are inversely proportional to the zero, second, and fourth power of the cycle number, respectively. As the cycle number increases gradually, $\pi_{\mathrm{II}}^{*}$(*m*) and $\pi_{\mathrm{III}}^{*}$(*m*) decrease and tend to 0, but $\pi_{I}^{*}$(*m*) keeps unchanged and is always equal to 1.

Furthermore, when the function π^*^(*m*) diminishes to zero, the VNDFBS approaches 0, and the normalized direction factors of bending stiffness converge to the limit, 1/*p*, demonstrating that the laminate achieved nearly constant bending stiffness. The value of π^*^(*m*) reflects how quickly the stacking sequence approaches constant bending stiffness. On the one hand, $\pi_{\mathrm{III}}^{*}$(*m*) ≈ [$\pi_{\mathrm{II}}^{*}$(*m*)]^2^, that is, the velocity of Pattern III approaching 0 is almost the square times that of Pattern II approaching 0. On the other hand, $\pi_{\mathrm{III}}^{*}$(*m*) ≈ $\pi_{\mathrm{II}}^{*}$(*m*^2^), namely, to achieve the same change ratio, the cycle number for Pattern II laminates is the square of that for Pattern II laminates, and the total thickness for Pattern II laminates is *m* times that for Pattern III laminates. So, to obtain the same uniformity in bending stiffness, the number of cycles of Pattern II laminates is the square of the number of cycles of Pattern II laminates.

## 3. Experiment Results and Discussion

**Figure 5:**
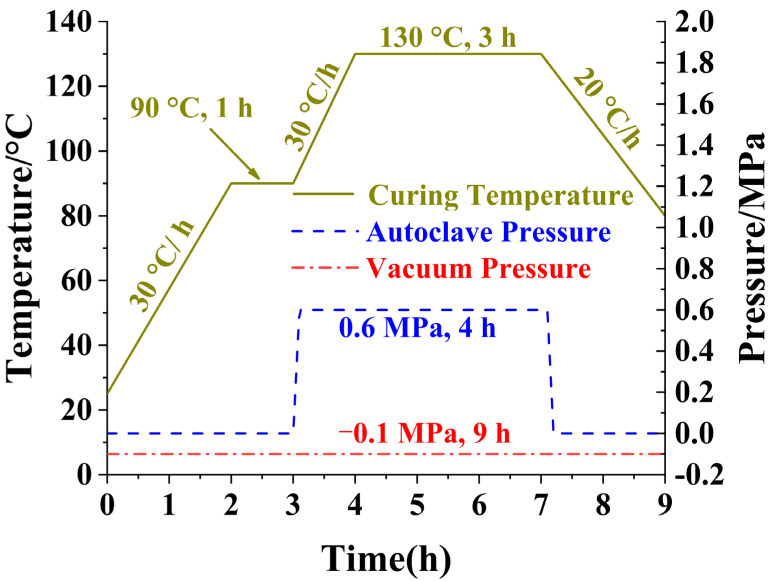
Curing temperature and pressure during the curing process by autoclave processing.

Laminates with dimensions of 440 mm × 240 mm and a thickness of 1.8 mm, 2.4 mm, or 3.6 mm were laid ply by ply with a T700S/602 prepreg (a single ply thickness of 0.15 mm). These were sealed within a vacuum bag and mold, and cured by autoclave processing with the following parameters: the curing temperature was set to 130 °C ± 5 °C, the vacuum bag pressure was maintained at −0.1 MPa (the vacuum pressure), and the autoclave pressure was held at 0.6 MPa, and more detailed information is shown on Figure 5. The mechanical properties of T700S/602 unidirectional laminates are shown in Table 1.

**Figure 6:**
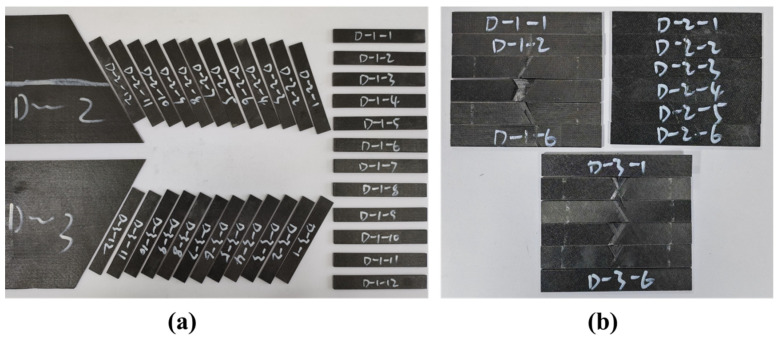
Typical cuboid specimens before (**a**) and after (**b**) the three-point bending test.

According to ASTM D 7264-2015 [40], the laminates were cut into the cuboid specimens at different orientations with varied dimensions, 85 mm × 12.5 mm × 1.8 mm for 12 plies (Stacking-C and Stacking-D), 100 mm × 12.5 mm × 2.4 mm for 16 plies (Stacking-A and Stacking-B), and 150 mm × 12.5 mm × 3.6 mm for 24 plies (Stacking-E and Stacking-F), respectively. They were subjected to the three-point bending test to measure the required bending moduli and strength, and six specimens were tested to obtain the average values. In the bending test, the loading speed was set to 1 mm/min. The support spans were 61 mm for Stacking-C and Stacking-D, 77 mm for Stacking-A and Stacking-B, and 121 mm for Stacking-E and Stacking-F, respectively. Figure 6 shows the typical cuboid specimens before and after the test.

### 3.1. Four-Angle Ply

Two *π*/4 quasi-isotropic laminates, [(45/−45/0/90)_S_]_2_ and [45/−45/0/90]_2S_, were manufactured, and the detailed information of the laminates is listed in Table 2, which presents the measured values of the bending moduli for four different directions as well.

The calculation shows that the two laminates’ mean values of bending moduli were nearly identical to each other, respectively. The range of bending moduli of Stacking-A was from 35.4 GPa to 48.2 GPa, and the interval length was obviously smaller than that of Stacking-B, which ranged from 30.9 GPa to 53.2 GPa. The difference in the bending moduli in Pattern II was much larger than that of Pattern III, and laminates with Pattern III exhibited bending moduli closer to uniformity. This was consistent with the results of the comparison between Equations (25) and (26) or between the lines in Figure 4a. Interestingly, the same rule was applied to strength, as shown in Table 2. For Pattern III, the difference between the maximum and minimum strength values was 100 MPa, while for Pattern II, the difference was 155 MPa.

The effective bending modulus, *E_f_*, of a laminate in the reference direction is as follows [41]:

(27) $$E_{f}=\frac{12}{d_{11}\delta^{3}}$$

where *d*_11_ is the first element of the bending compliance matrix. If the reference coordinate of the stacking sequence is rotated to a certain angle, the effective bending modulus in that orientation could be calculated.

**Figure 7:**
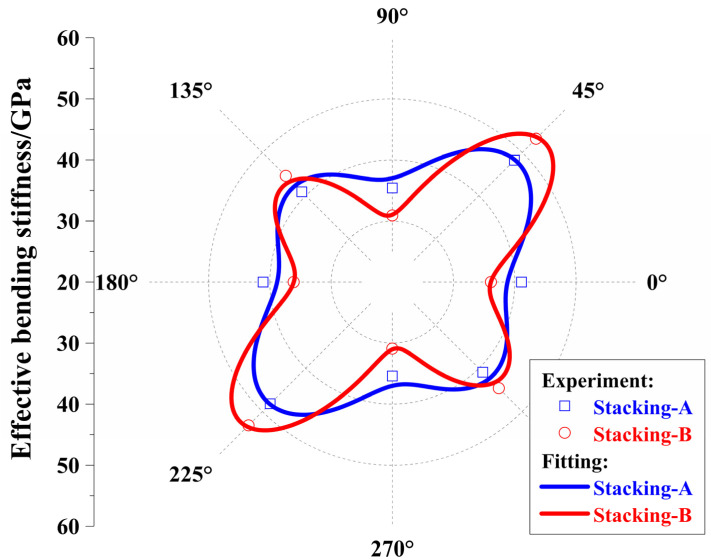
Effective bending modulus with regard to four-angle laminates from experiment and fitting.

For each laminate, the bending modulus of a 45° direction was the largest value, and that of a 90° direction was the smallest value, which is the same as the calculation values. Based on the experimental values of bending moduli, the optimal properties of the unidirectional composite materials, T700S/602, are obtained by Equation (27) and the genetic optimization algorithm [42]. Unlike other fitting methods, the bending stiffness and material parameters in this fitting process must be matched with the theory of classical composite mechanics. The objective function aims to minimize the discrepancy between the fitted bending moduli curve and experimental data of bending moduli at different angles, with the parameters being optimized corresponding to material parameters of unidirectional carbon-fiber composites. The corresponding bending moduli verse orientations are drawn in the polar coordinate, as shown in Figure 7, and the experimental results match well with those according to the classical lamination theory.

### 3.2. Three-Angle Ply

To verify the above conclusions further, four *π*/3 quasi-isotropic laminates, such as [(60/0/−60)_S_]_2_, [60/0/−60]_2S_, [(60/0/−60)_S_]_4_, and [60/0/−60]_4S_, were manufactured and indicated as Stacking-C, Stacking-D, Stacking-E, and Stacking-F in turns. The first two laminates, Stacking-C and Stacking-D, had 12 plies with a total thickness of 1.8 mm and the cycle number 2; the last two laminates, Stacking-E and Stacking-F, had 24 plies with a total thickness of 3.6 mm and the cycle number 4, which are depicted in Table 3.

Stacking-C had a bending modulus range of 37.3 GPa to 52.6 GPa, significantly narrower than Stacking-D’s range of 30.7 GPa to 65.7 GPa. The bending modulus of Stacking-E spanned from 40.9 GPa to 45.8 GPa, exhibiting a much narrower spread than Stacking-F, which ranged from 35.7 GPa to 51.1 GPa. These experimental data align with those for four-angle plies and the theoretical derivations. Equation (9) can be approximately simplified as follows:

(28) $$\eta^{*}(j)≅\frac{12}{n^{3}}\sum_{i=1}^{2m}{\left(h_{a_{ij}}^{*}\right)}^{2}$$

Regardless, the value of *j*, as η^*^(*j*), tends to approach the limit, 1/*p*, the more uniform the bending stiffness is. Stacking-C can be written as [60/0/−60/−60/0/60]s, and Stacking-D can be written as [60/0/−60/60/0/−60]s. Since both are symmetric laminates, only the first six plies need to be considered. For both laminates, the following conditions are satisfied: the first 60° ply is farther from the mid-plane than the first 0° ply, and both are farther than the first −60° ply. However, in Stacking-C, the second 60° ply is farther from the mid-plane than the second 0° ply, and both are farther than the second −60° ply. In Stacking-D, the second 60° ply is closer to the mid-plane than the second 0° ply, and both are closer than the second −60° ply. Considering Equation (28), the bending stiffness among the three angles in Stacking-C is more uniform compared to that in Stacking-D.

**Figure 8:**
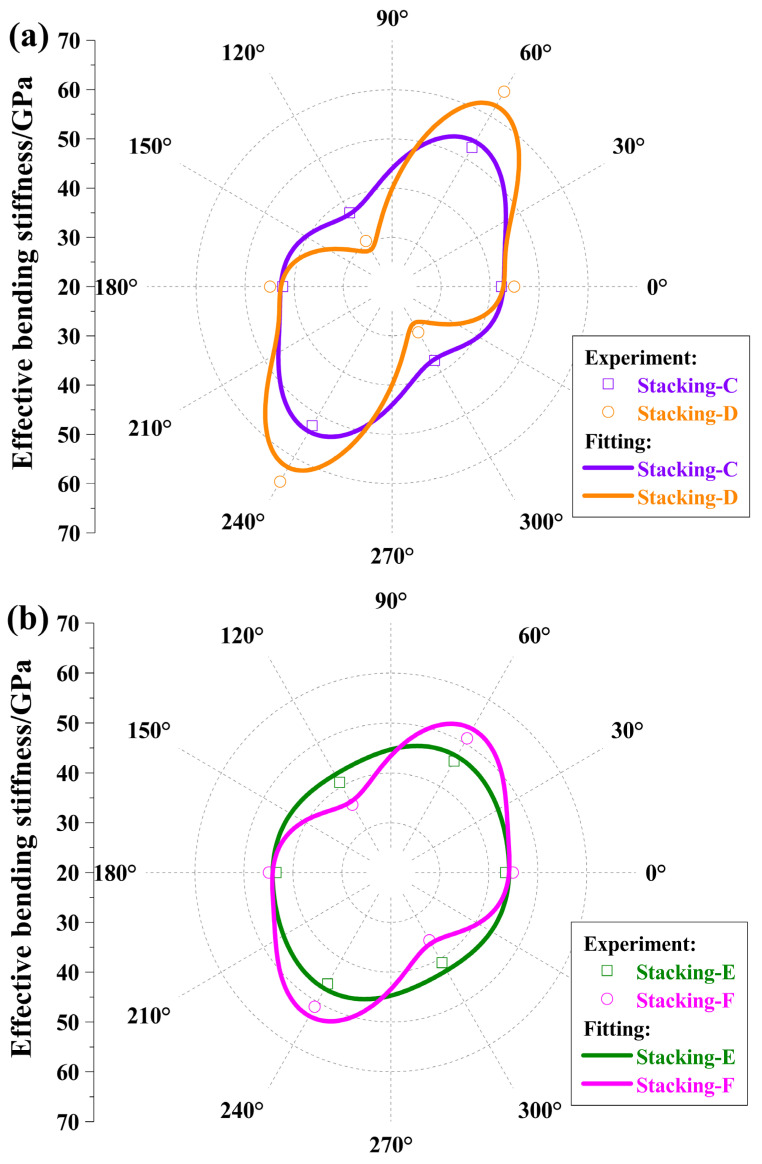
Effective bending moduli with regard to three-angle laminates from experiment and fitting. (**a**) *m* = 2, experimental data and fitting curves for Stacking-C and Stacking-D; (**b**) *m* = 4, experimental data and fitting curves for Stacking-E and Stacking-F.

For the four laminates, the bending stiffness values at the 60° direction are the largest and those at −60° direction are the smallest, which follows from the theoretical prediction. Similarly, in the four-angle ply, the unidirectional composite materials’ optimal or fitting property parameters were achieved, which were almost consistent with that in Table 1. The experimental and fitting values of the bending moduli are exhibited in Figure 8.

The average values of the bending moduli for each laminate were 44.0 GPa, 47.1 GPa, 43.4 GPa, and 43.9 GPa in turn, except that the value for Stacking-D was slightly higher than that for the others.

Comparing two rows in Table 3, between Stacking-C and Stacking-E or Stacking-D and Stacking-F, as the cycle number grows, the maximum bending modulus decreases, and the minimum increases gradually. In other words, the bending moduli tend to be more uniform in either Pattern II or Pattern III. Furthermore, the curve and point values corresponding to Stacking-C were nearly identical to those of Stacking-F. That is, based on the same basic laminate, Pattern III almost had the same bending moduli as Pattern II, which nearly had the square cycle number of Pattern III. This verifies the preceding result for the VNDFBS of Pattern III with the cycle number *m* = *l* was approximately identical to that of Pattern II with the cycle number *m* = *l*^2^, which is especially useful in the fields where weight loss is an urgent requirement, such as space mirrors. Additionally, we found that for Pattern III, the bending strength also became uniform, as shown in Table 3. This can be applied to fields where uniformity in bending strength is required.

## 4. Conclusions

We defined the normalized direction factors of bending stiffness based on the normalized geometric factors of bending stiffness. Then, by theoretical and experimental methods, two common stacking patterns, I ([(*θ*_1_)*_m_*/(*θ*_2_)*_m_*/…/(*θ_p_*)*_m_*]_S_) and II ([(*θ*_1_/*θ*_2_/…/*θ_p_*)*_m_*]_S_), and our proposed new stacking pattern, Pattern III ([(*θ*_1_/*θ*_2_/…/*θ_p_*)_S_]*_m_*), were considered to efficiently compare their uniformity in bending stiffness. As the cycle number grows, the uniformity of bending stiffness increases gradually, and the speed of Pattern III approaching the constant bending stiffness is faster than that of Patterns I and II. To achieve nearly identical bending stiffness uniformity, only 1/*m* (*m* is the cycle number) of the number of plies for Pattern II is needed for Pattern III. Moreover, Pattern III has a more uniform bending strength. So, laminates with Pattern III would be more suitable for space mirrors and other similar environments requiring fully isotropic laminates. At the same time, we can further extend the method of NDFBSs and VNDFBSs to obtain the stacking pattern with the more uniform bending stiffness.

## Figures and Tables

**Table 1:** Material parameters of unidirectional carbon-fiber composite.

Material	*E*_1_/GPa	*E*_2_/GPa	*G*_12_/GPa	*ν*_12_
T700/602	115	8.36	4.56	0.30

**Table 2:** Laminate information and experimental results for the *π*/4 quasi-isotropic laminates.

Serial Number	Pattern	Stacking Pattern	Specimen Size/mm	Number of Plies	Angle /°	*E_f_*/GPa		*σ_f_*/MPa	
						Mean	Std	Mean	Std
Stacking-A	III	[(45/−45/0/90)_S_]_2_	100 × 12.5 × 2.4	16	0	41.1	1.5	788	25.4
					45	48.2	1.7	691	67.0
					90	35.4	1.9	791	30.9
					−45	40.9	1.4	738	37.1
Stacking-B	II	[45/−45/0/90]_2S_	100 × 12.5 × 2.4	16	0	36.1	2.0	805	34.8
					45	53.2	2.6	798	76.6
					90	30.9	1.5	650	22.4
					−45	44.6	2.0	760	58.4

**Table 3:** Information on stacking patterns and experimental results of the *π*/3 quasi-isotropic laminates.

Serial Number	Pattern	Stacking Pattern	Specimen Size/mm	Number of Plies	Angle /°	*E_f_*/GPa		*σ_f_*/MPa	
						Mean	Std	Mean	Std
Stacking-C	III	[(60/0/−60)_S_]_2_	85 × 12.5 × 1.8	12	0	42.3	1.0	997	45.9
					60	52.6	2.5	766	77.3
					−60	37.3	1.6	968	68.2
Stacking-D	II	[60/0/−60]_2S_	85 × 12.5 × 1.8	12	0	44.9	2.5	1087	86.7
					60	65.7	4.0	920	59.4
					−60	30.7	1.8	806	27.8
Stacking-E	III	[(60/0/−60)_S_]_4_	150 × 12.5 × 3.6	24	0	43.4	0.7	927	93.9
					60	45.8	1.0	730	46.6
					−60	40.9	1.4	948	39.5
Stacking-F	II	[60/0/−60]_4S_	150 × 12.5 × 3.6	24	0	44.9	5.1	883	113.8
					60	51.1	1.7	810	117.0
					−60	35.7	2.4	692	61.4

## Data Availability

The original contributions presented in this study are included in the article. Further inquiries can be directed to the corresponding authors.
